# Efficacy of Urethral Sphincter Botulinum Toxin A Injection in Patients with Spinal Cord Injury with Dysuria: A Retrospective Cohort Study

**DOI:** 10.3390/toxins16080336

**Published:** 2024-07-31

**Authors:** Wan-Ru Yu, Jing-Hui Tian, Hann-Chorng Kuo

**Affiliations:** 1 Department of Nursing, Hualien Tzu Chi Hospital, Buddhist Tzu Chi Medical Foundation, Hualien 97002, Taiwan; wanzu666@gmail.com; 2 Department of Urology, Hualien Tzu Chi Hospital, Buddhist Tzu Chi Medical Foundation and Tzu Chi University, 707, Section 3, Chung Yang Road, Hualien 97002, Taiwan; 3 Voiding Dysfunction Therapeutic and Research Center, Hualien Tzu Chi Hospital, Buddhist Tzu Chi Medical Foundation, Hualien 97002, Taiwan; luckysweet999@gmail.com; 4 Department of Urology, School of Medicine, Tzu Chi University, Hualien 97004, Taiwan

**Keywords:** neurogenic bladder, botulinum toxin, urethral injection, voiding dysfunction, spinal cord injury

## Abstract

Spinal cord injury (SCI) often leads to neurogenic lower urinary tract dysfunction, causing dysuria and affecting patients’ well-being. This study aimed to evaluate the efficacy of a urethral sphincter botulinum toxin A (BoNT-A) injection in patients with SCI and dysuria. This was a retrospective study including 118 patients with SCI who underwent a urethral BoNT-A injection following a standardized protocol for refractory voiding dysfunction. The protocol involved injecting BoNT-A into the urethral sphincter under cystoscopic guidance. Patient demographics, bladder condition parameters, and treatment outcomes were analyzed. Logistic regression and receiver operating characteristic curve analyses were performed to identify predictors of treatment success. Of the 118 patients, 71 (60.1%) showed satisfactory treatment outcomes after the injection. Post-injection status, bladder management, and injection frequency varied significantly among patients with satisfactory and unsatisfactory treatment outcomes. Age, bladder compliance, intravesical pressure, and bladder contractility were indicators of satisfactory outcomes. The first sensation of bladder filling of ≤263 mL, intravesical pressure of ≤28, and bladder contractility index of ≥14 were highly correlated with satisfactory outcomes. A urethral sphincter BoNT-A injection shows promise in managing dysuria in patients with SCI. Understanding bladder condition parameters and patient demographics helps optimize patient selection for this intervention. Further studies are needed to validate these findings and refine treatment protocols.

## 1. Introduction

Spinal cord injury (SCI) disrupting the neural pathways between the brain, spinal cord, and lower urinary tract (LUT) is a life-threatening condition that results in long-term disability, leading to neurogenic lower urinary tract dysfunction, causing detrusor overactivity or detrusor sphincter dyssynergia (DSD), and significantly impacting patients’ quality of life (QoL) and mental health [1,2,3]. These conditions result in frequent, urgent urination, incontinence, and urinary retention, complicating the management of bladder function in SCI patients [4].

Effective management of DSD to prevent urological complications in SCI patients is crucial [5,6]. Botulinum toxin A (BoNT-A) injections into the urethral sphincter have emerged as a promising treatment for refractory voiding dysfunction in patients with SCI and DSD [7,8]. BoNT-A works by inhibiting acetylcholine release at the neuromuscular junction, reducing striated muscle hypertonicity and improving voiding efficiency [9,10]. However, the initial experience of BoNT-A injection into the urethral sphincter was found to have a limited improvement of the quality of life due to exacerbated urinary incontinence and incomplete bladder emptying [11]. Real-world efficacy still has to be explored. This study aimed to evaluate the efficacy of a urethral sphincter BoNT-A injection in patients with SCI and dysuria. By analyzing a large cohort of patient demographics, bladder condition parameters, and treatment outcomes, we might identify predictors of treatment success and optimize patient selection for this intervention.

## 2. Results

Of the 1066 patients with SCI, 118 who received the urethral BoNT-A injection (men: 94, women: 24) with a mean duration of SCI over 20 years were included in this study, 71 (60.1%) showed satisfactory treatment outcomes after a urethral BoNT-A injection. Significant differences in the “current bladder management”, “status after urethral BoNT-A injection”, and “urethral botulinum injection times” were observed between the satisfactory and unsatisfactory treatment outcome groups. Furthermore, no significant differences in gender, SCI level, autonomic dysreflexia (AD), completeness, hand function, current urination, voiding condition at baseline, cystometry results, and pressure flow analysis were observed between the two groups (Table 1).

Regarding the bladder condition parameters, a significant early first sensation of bladder filling (FSF) was observed in the satisfactory treatment outcome group compared with that in the unsatisfactory treatment outcome group (135.9 ± 74.2 vs. 171.4 ± 102.3, *p* = 0.044) (Table 2).

The area under the curve (AUC) revealed a higher specificity for age, ultrasound bladder capacity (US), bladder compliance at capacity, maximal intravesical pressures (Pves), and bladder contractility index (BCI), whereas the sensitivity for FSF, full sensation (FS), maximal detrusor pressure at maximal flow (Pdet), voided volume, post-voiding residual (PVR), and voiding efficiency index (VE) was greater (Table 3).

Logistic regression analysis revealed that FSF of ≤263 mL, maximal Pves of ≤28, and BCI of ≥14 were predictors of satisfactory treatment outcomes after urethral BoNT-A injection in patients with SCI (Table 4).

## 3. Discussion

This study evaluates the efficacy of urethral sphincter BoNT-A injections in managing dysuria among patients with SCI. It indicates that 60.1% of the patients demonstrated satisfactory treatment outcomes, showcasing the potential of this intervention. These results underscore the effectiveness of BoNT-A in improving voiding dysfunction, and more factors should be considered to achieve a satisfactory outcome [12] and identify specific bladder condition parameters—such as the first sensation of bladder filling, intravesical pressure, and bladder contractility index—that correlate strongly with positive treatment outcomes. This study advances the field by introducing novel predictors for treatment success, which have not been reported in previous literature focusing on neurogenic bladder with a urethral sphincter BoNT-A injection [4,13].

SCI is characterized by a disruption of neural control, resulting in the disruption or alteration of nerve signals that coordinate bladder and sphincter activity and DSD develops [14]. DSD is a challenging aspect of neurogenic bladder dysfunction in patients with SCI. Traditional treatments, such as catheterization and pharmacotherapy, offer temporary relief but may not address dysuria comprehensively [15]. The mechanism of action of BoNT-A in the urinary bladder involves inhibiting the release of acetylcholine, a neurotransmitter responsible for muscle contraction, at the nerve terminals. When BoNT-A is injected into the urethral sphincter, it is endocytosed into the nerve endings and blocks the release of acetylcholine, thereby inducing temporary paralysis of the sphincter muscle, reducing sphincter tone, and facilitating bladder emptying [9], potentially alleviating dysuria symptoms in patients with SCI [16]. However, previous studies have shown that urethral BoNT-A injection has less favorable treatment outcomes in patients with SCI and DSD than detrusor BoNT-A injection in patients with neurogenic detrusor overactivity and DSD, and patients still must empty their bladder by clean intermittent self-catheterization (CISC). Furthermore, the frequency of urinary incontinence episodes usually increases. Therefore, after careful consideration, surgical procedures have been suggested to improve spontaneous voiding or QoL for severely tight external sphincter or obstruction [17] in the second year after injury [18]. In other words, bladder symptoms and satisfaction are indeed affected by various factors [19]. Thus, it is necessary to construct a customized voiding intervention, long-term care, and a sense of self and hope for patients’ future based on their lives before injury [20]. Furthermore, a comprehensive personalization assessment would be indispensable.

In patients with SCI, lower urinary tract dysfunction is a predominant issue that significantly impacts their QoL and causes multiple complications [20,21,22]. However, a comprehensive urological intervention for SCI has not been well established. Therefore, the high proportion (>70%) of frequent urinary tract infections (UTIs) in patients with SCI with dysuria should be taken seriously, even with improvement in the voiding condition after urethral BoNT-A injection. A previous study has shown that <10% of patients would achieve a good bladder status, indicating that a suitable bladder treatment might not be easy to approach, and repeat treatments and long-term follow-up should be the primary focus. Additionally, AD, a potentially life-threatening complication in more than 70% of patients with SCI, is a secondary complication and a severe and chronic problem [23]. Urethral BoNT-A injection can reduce dysuria severity, decrease DSD, improve bladder emptying, and decrease AD. However, an extensive assessment should be performed to tailor individualized interventions, considering the cost, convenience, long-term care with specialized units, and the nurse practitioner role [24,25].

The strengths of this study lie in providing insight into the predictors of treatment satisfaction, helping clinicians identify candidates for a urethral BoNT-A injection, and understanding the characteristics of patients with SCI. However, this study has some limitations. The sample size may not adequately reflect the diverse population of patients with SCI. Furthermore, this was a retrospective study. Thus, the generalizability of its findings may be limited. Despite its efficacy, a urethral BoNT-A injection has certain limitations and potential adverse effects, including transient urinary retention, UTIs, and the risk of urinary incontinence. Therefore, careful patient selection and close monitoring are necessary to minimize these risks and maximize treatment benefits. Thus, further studies with larger sample sizes and longer follow-up periods are needed to validate these findings and assess the long-term efficacy and safety of BoNT-A injections.

## 4. Conclusions

A repeat urethral sphincter BoNT-A injection is a minimally invasive therapeutic option for effectively managing dysuria in patients with SCI. Specific bladder condition parameters such as the FSF, intravesical pressure, and BCI are critical in predicting treatment success. While our findings are promising, further research is needed to refine patient selection criteria and optimize treatment protocols to maximize therapeutic benefits and improve the quality of life for patients with SCI and dysuria. Future studies should also explore the long-term outcomes and safety of repeated BoNT-A injections in this patient population.

## 5. Materials and Methods

### 5.1. Study Design and Patients

This was a retrospective study of 1066 consecutive patients diagnosed with SCI and voiding dysfunction between 2011 and 2022. The inclusion criteria were patients with SCI and voiding dysfunction refractory to medical treatment who received urethral BoNT-A injections (onabotulinumtoxin A, Allergan, Irvine, CA, USA) at a dose of 100 U into the urethral sphincter. All patients underwent video urodynamic studies (VUDS) to confirm the underlying pathophysiology of neurogenic lower urinary tract dysfunction before the BoNT-A injection.

### 5.2. Data Collection

Data were collected from one medical center, where the same urology team performed all treatments. VUDS parameters, including PVR, FSF, FS, US, bladder compliance, maximal Pves, maximal Pdet, maximal flow rate (Qmax), and voided volume, were collected. Additionally, the VE index, BCI, and bladder outlet obstruction index (BOOI) were measured. The terminology used in this study was based on the International Continence Society recommendations [26].

### 5.3. Treatment

BoNT-A was reconstituted with 4 mL of normal saline to achieve a concentration of 25 U/mL. Approximately 100 U of BoNT-A was injected into the transurethral sphincter under intravenous general anesthesia. Based on a previous study, each 1 mL of BoNT-A solution was injected transurethrally four points [27].

After BoNT-A injections, a Foley catheter was inserted overnight. The patients were instructed to return after 3 months to report voiding conditions. The effects on urethral sphincter function were observed 2–3 days after injection, with the maximum effect potentially seen after 2 weeks [28]. Patients with detrusor underactivity and a large PVR were advised to use the Crede maneuver or abdominal straining for voiding, with CISC recommended over a Foley catheter. Regardless of the ability to urinate via percussion or abdominal pressure, CISC was encouraged until the PVR decreased to <50% of the voided volume. Antibiotics were routinely administered for 3 days after injection to prevent UTIs.

### 5.4. Treatment Outcomes

The effectiveness of a BoNT-A injection was evaluated based on self-reported voiding improvement and satisfaction 6 months after treatment. Improvement was categorized as no change, mild (<50% reduction in CISC difficulty and frequency), moderate (no difficulty, CISC frequency of <25%), and significant (no voiding difficulty, no need for CISC). No change and mild improvement indicated unsatisfactory outcomes, whereas a moderate-to-significant improvement indicated satisfactory outcomes.

### 5.5. Ethical Considerations

The potential adverse effects of a BoNT-A injection were explained to all patients before treatment. The study was approved by the Hualien Tzu Chi Hospital’s Research Ethics Committee (IRB: 110-033-B). As this was a retrospective study, the Research Ethics Committee of Hualien Tzu Chi Hospital waived informed consent requirements. All methods used in this study were conducted in accordance with relevant guidelines and regulations.

### 5.6. Data Analysis

Factors influencing treatment outcomes were analyzed using the R software. Logistic regression and receiver operating characteristic curve analyses were performed to identify the predictors of treatment success, and the area under the curve (AUC) was calculated. A *p*-value of <0.05 indicated statistical significance.

## Figures and Tables

**Table 1:** Baseline characteristics between different treatment outcomes after urethral botulinum toxin A injection therapy in patients with spinal cord injury (*n* = 118).

	Unsatisfactory Outcome Group (*n* = 47)	Satisfactory Outcome Group (*n* = 71)	*p*-Value
Gender			
*Female:Male*	8:38 (17%:83%)	16:55 (22.5%:77.5%)	0.466
Age	48.3 ± 15.0	47.4 ± 17.6	0.780
Duration of SCI	28.5 ± 9.9	26.5 ± 9.7	0.314
SCI level			
*Cervical*	17 (36.2%)	34 (47.9%)	0.416
*Thoracic*	19 (40.4%)	24 (33.8%)	
*Lumbar sacral/infra-sacral lesion*	11 (23.4%)	13 (18.3%)	
Autonomic dysreflexia	36 (76.6%)	51 (71.8%)	0.565
Completeness of SCI			
*Complete*	35 (74.5%)	45 (63.4%)	0.207
*Incomplete*	12 (25.5%)	26 (36.6%)	
Hand function			
*Normal*	39 (83%)	50 (70.4%)	0.207
*Partial*	3 (6.4%)	12 (16.9%)	
*Incapable*	5 (10.6%)	9 (12.7%)	
Current urination			
*Dysuria*	31 (66%)	53 (74.6%)	0.169
*UUI*	2 (4.3%)	6 (8.5%)	
*Urine retention*	14 (29.8%)	12 (16.9%)	
Current bladder management			
*Self-voiding reflex*	5 (10.6%)	4 (5.6%)	0.009 *
*Self-voiding percussion*	18 (38.3%)	46 (64.8%)	
*Straining voiding, abdominal pressure*	11 (23.4%)	13 (18.3%)	
*CISC, UUI*	0	1 (1.4%)	
*CISC, no UUI*	5 (10.6%)	5 (7.0%)	
*Suprapubic or urethral catheter, no UUI*	8 (17%)	2 (2.8%)	
Status after urethral BoNT-A injection			
*Doing well*	1 (2.1%)	6 (8.5%)	0.015 *
*Frequent UTI*	33 (70.2%)	56 (78.9%)	
*Urinary incontinence*	1 (2.1%)	2 (2.8%)	
*Difficult self-voiding*	12 (25.5%)	7 (9.9%)	
Urethral botulinum injection times			
1 *to* 2 *times*	46 (97.8%)	64 (90.2%)	0.001 *
*More than three times*	1 (2.1%)	7 (9.9%)	
Cystometry			
*Stable or Hypersensitive bladder*	3 (6.4%)	2 (2.8%)	0.202
*Detrusor overactivity*	29 (61.7%)	58 (81.7%)	
*Detrusor underactivity*	15 (31.9%)	11 (15.5%)	
Pressure flow analysis			
*Low pressure, high flow, non-obstruction*	1 (2.1%)	0	0.565
*Low pressure, low flow, non-obstruction*	5 (10.6%)	8 (11.3%)	
*High pressure, high flow, obstruction*	0	1 (1.4%)	
*High pressure, low flow, obstruction*	16 (34%)	34 (47.9%)	
*Low pressure, low flow, obstruction*	24 (51.1%)	27 (38%)	
*Equivocal*	1 (2.1%)	1 (1.4%)	

SCI: spinal cord injury, UUI: urge (urgency) urinary incontinence, CISC: clean intermittent self-catheterization, UTI: urinary tract infection, *: *p* < 0.05.

**Table 2:** Baseline video urodynamic study parameters of bladder condition between different treatment outcomes in patients with spinal cord injury (*n* = 118).

	Unsatisfactory Outcome Group (*n* = 47)	Satisfactory Outcome Group (*n* = 71)	*p*-Value
Post-voiding residual	272.2 ± 205.5	234.7 ± 162.2	0.272
First sensation of filling	171.4 ± 102.3	135.9 ± 74.2	0.044 *
Full sensation	242.8 ± 119.3	205.4 ± 108.7	0.081
Ultrasound bladder capacity	272.6 ± 136.6	235.3 ± 126.6	0.132
Bladder compliance at capacity	61.3 ± 76.9	45.8 ± 64.7	0.257
Maximal intravesical pressure_cmH_2_O	57.0 ± 36.0	51.7 ± 30.5	0.390
Maximal detrusor pressure at Qmax_cmH_2_O	26.2 ± 23.2	32.8 ± 22.6	0.126
Maximal flow rate	3.4 ± 4.6	3.5 ± 3.9	0.927
Voided volume	84 ± 124.6	53.1 ± 74.8	0.132
Voiding efficiency index (VE)	0.26 ± 0.33	0.23 ± 0.28	0.582
Bladder contractility index (BCI)	43.7 ± 32.7	50.7 ± 30.2	0.236
Bladder outlet obstruction index (BOOI)	19.2 ± 25.1	25.7 ± 23.9	0.161

*: *p* < 0.05.

**Table 3:** Specificity and sensitivity under the area under the curve.

	AUC	95% CI	Cutoff	Sensitivity	Specificity
Age	0.516	0.411–0.622	≤38.5	0.394	0.745
FSF	0.583	0.477–0.689	≤263	0.972	0.170
FS	0.595	0.489–0.702	≤252	0.746	0.447
US	0.591	0.482–0.700	≤271	0.553	0.704
Compliance at capacity	0.596	0.491–0.701	≤15.3	0.408	0.851
Maximal Pves_cmH_2_O	0.531	0.424–0.639	≤28	0.239	0.894
Maximal Pdet_cmH_2_O	0.590	0.483–0.697	≥14	0.817	0.383
Maximal flow rate	0.526	0.422–0.629	≥3	0.493	0.617
Voided volume	0.520	0.413–0.627	≤53	0.704	0.426
PVR	0.546	0.436–0.656	≤320	0.761	0.362
VE	0.504	0.398–0.609	≥0.35	0.732	0.362
BCI	0.569	0.460–0.678	≥14	0.298	0.901
BOOI	0.582	0.475–0.688	≥18	0.592	0.574

AUC: area under the curve, FSF: first bladder sensation during filling, FS: full sensation, US: ultrasound bladder capacity, maximal Pves: maximal intravesical pressure, maximal Pdet: maximal detrusor pressure at Qmax, PVR: post-voiding residual, VE: voiding efficiency index, BCI: bladder contractility index, BOOI: bladder outlet obstruction index.

**Table 4:** Logistic regression for predicting satisfactory treatment outcome effect factors after a urethral botulinum toxin A injection in patients with spinal cord injury.

Predictor Factor	Cutoff	β	OR 95% CI	*p*-Value
First sensation of filling	≤263	1.856	1.142–35.869	0.035
Maximal intravesical pressures (Pves)	≤28	3.888	1.182–12.789	0.025
Bladder contractility index	≥14	3.533	1.354–9.222	0.010
Constant		0.346		0.021

## Data Availability

Data are available upon request from the corresponding authors.
